# Phenotypical and ultrastructural features of Oct4-positive cells in the adult mouse lung

**DOI:** 10.1111/jcmm.12295

**Published:** 2014-06-03

**Authors:** Celimene Galiger, Sawa Kostin, Anita Golec, Katrin Ahlbrecht, Sven Becker, Mihaela Gherghiceanu, Laurentiu M Popescu, Rory E Morty, Werner Seeger, Robert Voswinckel

**Affiliations:** a Department of Lung Development and Remodeling, Max-Planck-Institute for Heart and Lung ResearchBad Nauheim, Germany; b Core Lab for Molecular and Structural Biology, Max-Planck-Institute for Heart and Lung ResearchBad Nauheim, Germany; c “Victor Babes” National Institute for PathologyBucharest, Romania; d Department of Cellular and Molecular Medicine, “Carol Davila” University of Medicine and PharmacyBucharest, Romania

**Keywords:** Oct4, telocytes, stem cells, adult mouse lung

## Abstract

Octamer binding trascription factor 4 (Oct4) is a transcription factor of POU family specifically expressed in embryonic stem cells (ESCs). A role for maintaining pluripotency and self-renewal of ESCs is assigned to Oct4 as a pluripotency marker. Oct4 can also be detected in adult stem cells such as bone marrow-derived mesenchymal stem cells. Several studies suggest a role for Oct4 in sustaining self-renewal capacity of adult stem cells. However, Oct4 gene ablation in adult stem cells revealed no abnormalities in tissue turnover or regenerative capacity. In the present study we have conspicuously found pulmonary Oct4-positive cells closely resembling the morphology of telocytes (TCs). These cells were found in the perivascular and peribronchial areas and their presence and location were confirmed by electron microscopy. Moreover, we have used Oct4-GFP transgenic mice which revealed a similar localization of the Oct4-GFP signal. We also found that Oct4 co-localized with several described TC markers such as vimentin, Sca-1, platelet-derived growth factor receptor-beta C-kit and VEGF. By flow cytometry analyses carried out with Oct4-GFP reporter mice, we described a population of EpCAM^neg^/CD45^neg^/Oct4-GFP^pos^ that in culture displayed TC features. These results were supported by qRT-PCR with mRNA isolated from lungs by using laser capture microdissection. In addition, Oct4-positive cells were found to express Nanog and Klf4 mRNA. It is concluded for the first time that TCs in adult lung mouse tissue comprise Oct4-positive cells, which express pluripotency-related genes and represent therefore a population of adult stem cells which might contribute to lung regeneration.

## Introduction

Octamer binding trascription factor 4 (Oct4) is a transcription factor of POU family also known as Pou5f1 (Pou domain 5 transcriptor factor 1) [1,2]. It is well-established that Oct4 is responsible for the pluripotency of embryonic stem cells (ESCs) and for their maintenance in undifferentiated state [3–6]. Oct4 down-regulation causes the differentiation of ESCs in the three germ layers: endoderm, mesoderm and ectoderm which give rise to different organs and tissues in the body [3]. Oct4 has also been detected in adult tissues cells such as human peripheral blood mononuclear cells, bone marrow-derived mesenchymal stem cells and human cancer stem cells [7–10]. Several studies suggested a role of Oct4 in maintaining self-renewal in somatic stem cells [11,12]. Although Oct4 gene ablation in somatic stem cells revealed no abnormalities in tissue homoeostasis or regenerative capacity, there is increasing evidence that Oct4, even if it is expressed at low levels in somatic cells, is dispensable for the self-renewal and maintenance of somatic stem cells [13,14].

Recently, Oct4 was found to be expressed in telocytes (TCs) in adult human skeletal muscle cell niche [15]. Telocytes are a novel described interstitial (stromal) cell type which display distinct structural features, with long extensions up to 100 μm called telopodes [16,17]. The telopodes comprise thin fragments called podomers and more enlarged fragments called podoms [18]. The TCs form a 3-dimensional network with intercellular junctions [19]. These cells were found in various organs including pleura, epicardium, myocardium, endocardium, intestine, uterus, pancreas, mammary gland and in several others organs (for review see: [17]). Previous electron microscopic studies of the adult mouse lung have shown that the TCs are located in the perivascular and peribronchial spaces and extend to the alveolar compartment [20].

Telocytes express several markers including CD34, alpha-smooth muscle actin (α-SMA), Sca-1, platelet-derived growth factor receptor-beta (PDGFR-β), S-100, C-kit, vimentin and VEGF in human epicardium, human term placenta or skeletal muscle cell niche [15,21]. A role of TCs in inter-cellular signalling *via* paracrine secretion as well as by shed vesicles and exosomes has been suggested because of their distinguished architecture with thin and long telopodes [22]. Their presence in the microenvironment of stem cell niches as well as the expression of stem cell markers suggests a role of these cells in tissue regeneration [23].

In this study, we have identified Oct4 expressing cells in the adult mouse lung. These cells are present in the perivascular and peribronchial spaces corresponding to the localization of TCs identified by electron microscopy. In addition, Oct4-positive cells were found to express several described markers of TCs, such as vimentin, Sca-1, PDGFR-β, C-kit and VEGF. These results were supported by qRT-PCR with mRNA isolated from cell picking by using laser capture microdissection technique. By using Oct4-GFP reporter mice, we were able to isolate Oct4-positive cells that in long-term culture conspicuously displayed morphological and phenotypical features of TCs. In addition, given that Oct4-positive cells in adult mouse lung are EpCAM^neg^/CD45^neg^/Oct4-GFP^pos^ cells and on the basis of negative selection with EpCAM, we demonstrate that Oct4-positive cells are not epithelial cells.

## Materials and methods

### Animal for experimentations

For this study, adult 8 week-old wild-type mice and Oct4-GFP transgenic mice were used. Adult wild-type mice were bred in our animal facility and Oct4-GFP mice were purchased from Jackson Laboratory, Bar Harbor, ME USA bred and maintained in our facility.

### Western blot analysis

Proteins were isolated from lung tissue lysates. In brief, 20–30 mg of tissues were transferred into bead tubes and homogenized by using a precellys homogenizer (PEQLAB Biotechnologie GmbH, Erlangen, Germany) at 6500 r.p.m. for 1 min. in RIPA lysis buffer (cat# 89901; Thermo Scientific, Rockford, IL USA) with protease and phosphatase inhibitor cocktail (cat# 18161284; Thermo Scientific). After homogenization, the samples were centrifuged at 15,000 × g at 4°C for 30 min. The supernatant was collected in new tubes and a colorimetric protein assay kit (Bio-Rad protein assay kit: cat# 210003399, Munich, Germany) was used to measure the levels of proteins. Before electrophoresis, the samples were mixed with Laemmli buffer [375 mmol, SDS 10% (w/v), glycerol 50% (v/v), β-mercaptoethanol 12.5% (v/v), bromophenol blue 0.02% (v/v)] and heated at 95°C for 5 min. followed by centrifugation with a speed of 15,000 × g for 12 sec. before loading. The samples were run on 10% SDS-PAGE (375 mmol Tris/Cl, pH 8.9, 10% acrylamide, 0.20% SDS, 0.05% and 0.10% TEMED) for 75 min. with 80–100 V and transferred on nitrocellulose membrane (cat# S80209; Pall Corporation, Dreieich, Germany) for 75 min. with 100 V. The membranes were rinsed with TBS/T (tris buffered saline with 1% Tween 20), blocked in 5% milk (cat# M740; Sigma-Aldrich, St. Louis, MO, USA) and then incubated with mouse monoclonal Oct-3/4 (sc-5279; Santa Cruz, Dallas, TX, USA) antibody in 1:1000 dilution overnight at 4°C. After washing with TBS/T, the membranes were incubated 1 hr with a goat antimouse secondary antibody (1:3000). A Super Signal reagent (cat# 34096; Thermo Scientific) was used for developing the membrane. The bands were detected by the luminescent image analyser LAS-4000 mini (Fujifilm, Tokyo, Japan). After exposure, the membranes were washed in TBS/T, submerged in 25 ml of stripping buffer (cat# 21059; Thermo Scientific) and incubated for 1 hr at room temperature (RT) on a shaker plate. Then the membranes were blocked in the milk and the immunodetection protocol was repeated with a mouse monoclonal anti-β-actin antibody 1:5000 (cat# A5316; Sigma-Aldrich) and the appropriate secondary antibody.

### Lung isolation and tissue preparation

Mice were anaesthetized with isofluran. A large incision was made up to the neck of the mouse. Ten millilitre PBS was perfused through the right ventricle until the lungs were cleared of blood. After pulling back the skin above the head of the mouse to expose the throat, the rest of the rib cage and other surrounding tissues were carefully removed to expose the trachea. A solution of 4% paraformaldehyde (PFA) was injected into the trachea until the lungs inflate. The lungs were dissected and dropped into a conical tube filled with 15 ml of 4% PFA at 4°C overnight. Thereafter, the lungs were processed for paraffin embedding in a tissue processor (ASP200S; Leica Microsystems, Nussloch GmbH, Germany).

### Immunohistochemistry

Ten micrometre-thick paraffin-embedded tissue sections were placed at 65°C for 20 min., followed by routine deparaffinization and dehydration steps. To block endogenous peroxidase, the tissue sections were incubated with 2% bovine serum albumin (BSA) containing 0.5% NP-40 (Sigma-Aldrich) and 3% normal goat serum. The slices were stained overnight with mouse monoclonal Oct4 primary antibody diluted 1:50 in 2% BSA with 0.5% NP-40 (Sigma-Aldrich). The tissues were rinsed in PBS and incubated for 1 hr at RT with the appropriate biotin-labelled secondary antibody (M.O.M kit, cat# PK-2000; Vector Laboratories, Burlingame, ON, Canada) at 1:1000 dilution. After repeated washing steps in PBS, the tissue slides were incubated for 1 hr at RT with the Vecstain® ABC substrate (M.O.M kit). The slides were then washed three times in PBS and incubated with 10 mg 3,3′-diaminobenzidine (cat# D4168; Sigma-Aldrich) diluted in PBS solution with 30% H_2_O_2_ for 15–30 min. at RT. After counterstaining with 4′,6-diamidino-2-phenylindole (DAPI, Molecular Probes, Darmstadt, Germany), the slides were mounted with Mowiol (cat# 475904; Merck Chemical Ltd., Darmstadt, Germany).

### Immunofluorescence and confocal microscopy

After routine deparaffinization and dehydration, the sections were incubated for 10 min. at 37°C with 1% trypsin (Digest All, cat# 003008; Invitrogen, Darmstadt, Germany) for antigen unmasking. After washing with PBS, the tissues were blocked with 5% BSA for 1 hr at RT and incubated with the appropriate primary antibodies diluted in 3% BSA with 0.2% TritonX-100 in PBS overnight at 4°C. Mouse monoclonal Oct-3/4 (C-10), dilution 1:50 (sc5279; Santa Cruz), mouse monoclonal anti-vimentin, 1:500 (C 9080; Sigma-Aldrich), monoclonal anti-α-actin smooth muscle conjugated with Cy3 (α-SMA), 1:500 (C6198; Sigma-Aldrich), anti-PDGFR-α, 1:100 (ab90967; Abcam, Cambridge, UK), rabbit polyclonal PDGFR-β, 1: 100 (sc-432; Santa Cruz), rat antimouse Ly-6A/E/Sca-1, 1:100 (cat # 553333; BD Biosciences, Heidelberg, Germany), rabbit polyclonal C-kit, 1:100 (sc-168; Santa Cruz), rabbit polyclonal VEGF, 1:100 (sc-152; Santa Cruz) were used for the staining. The sections where then incubated with the appropriate secondary antibodies (1:500–1000) for 1 hr at RT, washed with PBS and counterstained with DAPI (1:1000) for 10 min. The slices were mounted with Mowiol and investigated with a Zeiss LSM 710 laser scanning confocal microscope.

### Flow cytometry and cell sorting

The lungs were isolated and filled up with 2 ml dispase (cat# 354235; BD Bioscience). After mincing with a sterile scalpel, the lung tissue was incubated with 2 μg/ml collagenase B (cat# 11088815001; Life Sciences, Darmstadt, Germany) and 0.001% DNAse (cat#18535; Serva Electrophoresis, Heidelberg, Germany) in DMEM for 15 min. at 37°C. The resulting cell suspension was filtered through a 100 μm and 40 μm cell strainers (cat# 352340; BD Falcon, Heidelberg, Germany), harvested by centrifugation and resuspended in cell blood lysis buffer (0.15 mol NH_4_Cl, 10 mmol KHCO_3_, 0.1 mmol EDTA). After washes in PBS, the cells were resuspended in FACS-buffer (PBS, 2 mmol EDTA, 25 mmol HEPES) at 1 million cells per 100 μl buffer and incubated for 20 min. on ice with a mixture of anti-EpCAM and anti-CD45 antibodies (e-Biosciences, Frankfurt, Germany). The cells were analysed with a BD LSR II flow cytometer by using flowJo7.6.4 software, or were sorted with a BD FACSAria III cell sorter.

### Cell culture

Oct4-positive cells were sorted based on GFP fluorescence. The GFP-positive cells were collected in FACS-tubes filled with α-MEM medium (cat# 41061; Invitrogen) containing 10% foetal calf serum supplemented with penicillin/streptomycin, insulin/transferrin/selenium (cat# 51500-56; Invitrogen), 2 mmol l-glutamine (cat# P04-80100; PAN Biotech, Aidenbach, Germany) and 0.0002% heparin (cat# H3149; Sigma-Aldrich). After centrifugation for 5 min. at 400 × g, the cells were resuspended in MEM medium and kept in culture chamber slides for 5 days in normoxic conditions.

### Embryonic stem cells

Embryonic stem cell line E14 was derived from inbred mouse strain 129/Ola. The cells were plated in culture chambers coated with 0.2% gelatin and were kept in basal medium supplemented with 20% foetal calf serum, 1.25% sodium puryvate, 1.25% non-essential amino acids, 1.25% penicillin/streptomycin, 0.0088% beta-mercaptoethanol, 0.01% leukaemia inhibitory factor (Gibco, Darmstadt, Germany) and co-cultured with feeders cells.

### Laser capture microdissection and quantitative PCR

Frozen, 10 μm-thick tissues sections were used for the microdissection. The tissues sections were placed in 70% ethanol for 60 sec. and directly passaged in sterile distilled water for 10 sec. The slides were then shortly stained with haematoxylin (cat# MHS16; Sigma-Aldrich), washed in sterile water and tap water for 10 sec. each. Rapid steps of rehydration with increasing concentrations of ethanol were done to prevent the slides to dry and to maintain the integrity of the RNA. The microdissected samples were collected in tube filled with 350 μl of RLT buffer (Qiagen, Hilden, Germany), vortexed for 30 sec. and RNA were isolated by using the RNeasy Plus Micro Kit (Qiagen® Kit, cat# 74034). The reverse transcription PCR (RT-PCR) was performed with a cDNA kit from Bio-Rad (iScript™ Select cDNA Synthesis Kit, cat# 170-8896). Primers used for amplification are shown in Table 1. mRNA levels of different genes were measured by qRT-PCR during 40 cycles of amplification and were expressed as ΔCt values compared to GAPDH housekeeping gene control. Similar PCR protocols were employed for isolated Oct4-positive cells and ESCs.

**Table 1:** Primers used for determination of mRNA expression levels by qRT-PCR

Primer names	Foward primer	Reverse primer
Oct-4	5′-caagttggcgtggagactttgc-3′	5′-ccccaaggtgatcctcttctgc-3′
Klf4	5′-tgtgactatgcaggctgtggc-3′	5′-ggccctgtcacacttctggc-3′
Nanog	5′-gaacgcctcatcaatgcctgc-3′	5′-tgttctcctcctcctcagggc-3
Sox2	5′-gggctctgtggtcaagtccg-3′	5′-cgctctggtagtgctgggc-3′
Vimentin	5′-gagatcgccacctacaggaa-3′	5′-tccatctctggtctcaaccg-3′
PDGFR-β	5′-agggggcgtgatgactagc-3′	5′-ttccaggagtgataccagctt-3′
Sca-1	5′-gcctgcaaccttgtctgag-3′	5′-cagactccatcagggtaggg-3′
VEGF	5′-ggagatccttcgaggagcactt-3′	5′-ggcgatttagcagcagatataagaa-3′
C-kit	5′-tgtaaggcctccaacgatgt-3′	5′-cagagtgtgggcctggattt-3′
GAPDH	5′-tgaggccggtgctgagtatgtcg-3′	5′-ccacagtcttctgggtggcagtg-3′

### Transmission electron microscopy

Transmission electron microscopy (TEM) was performed on small (1 mm^3^) lung tissue samples, which were processed according to a routine Epon-embedding procedure as previously described [20,24–26]. Briefly, mouse lung samples were fixed by immersion in 4% glutaraldehyde, post-fixed in 1% OsO_4_ and further processed for Epon embedding. Thin sections (60 nm) were double-stained with uranyl acetate and lead citrate and examined under a Morgagni 268 transmission electron microscope (FEI Company, Eindhoven, The Netherlands) at 80 kV. Digital electron micrographs were recorded with a MegaView III CCD using iTEM-SIS software (Olympus, Soft Imaging System GmbH, Münster, Germany).

## Results

### Oct4 is expressed in adult mouse lung tissues

**Fig. 1:**
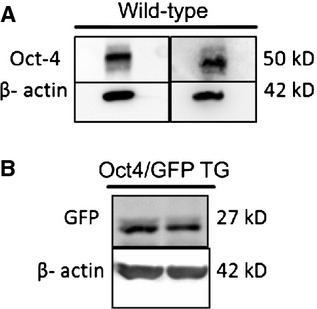
Western blots for Oct4 and GFP in adult wild-type and Oct4-GFP transgenic mice lung lysates. (**A**) Oct4 detection in two wild-type mouse lung lysates shows a band size of 50 kD (predicted molecular weight: 39–45 kD). (**B**) GFP detection in 2 Oct4-GFP transgenic mouse lung lysates shows a band size of 27 kD (predicted molecular weight: 27 kD).

Western blot analyses were performed to detect the expression of Oct4 in whole cell lung lysate. Samples from adult wild-type and Oct4-GFP transgenic mice were used. A band of 50 kD was detected by using the C-10 anti-Oct4 antibody in wild-type cell lysates (Fig. 1A) and a band of 27 kD was detected with a GFP antibody in transgenic cell lysates (Fig. 1B).

### Oct4 is expressed in peribronchial and perivascular cells and display TC features

**Fig. 2:**
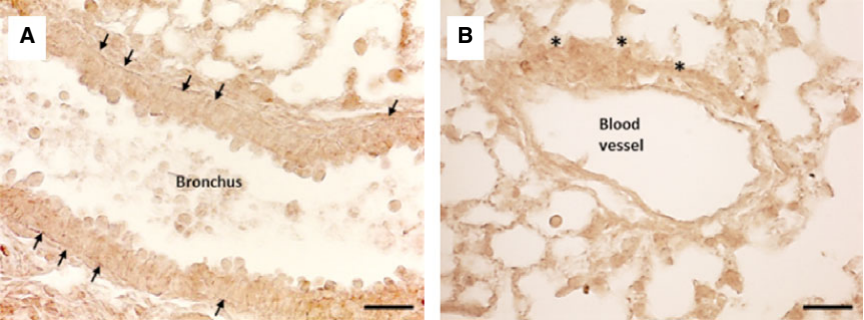
Immunohistochemical labelling of Oct4 in adult wild-type mouse lung. The DAB dark brown colour representing Oct4 expression is found in traceable amounts and is marked with arrows and asterisks. (**A**) Indicated with arrows are Oct4-positive cells circumscribing the bronchial epithelium, or with asterisks (**B**) around a blood vessel; bar = 50 μm

DAB staining and immunofluorescence on paraffin-embedded tissues sections were carried out to determine the localization and the labelling pattern of the Oct4 signal. Both, immunohistochemical and immunofluorescent analyses revealed the presence of Oct4-positive cells in the peribronchial (Fig. 2A) and in the perivascular areas (Fig. 2B). The intracellular distribution of Oct4 was observed in cytoplasmic pattern. Similar pattern of Oct4-positivity by using the C-10 anti-Oct4 antibody has previously been observed in adult stem cells [27].

**Fig. 3:**
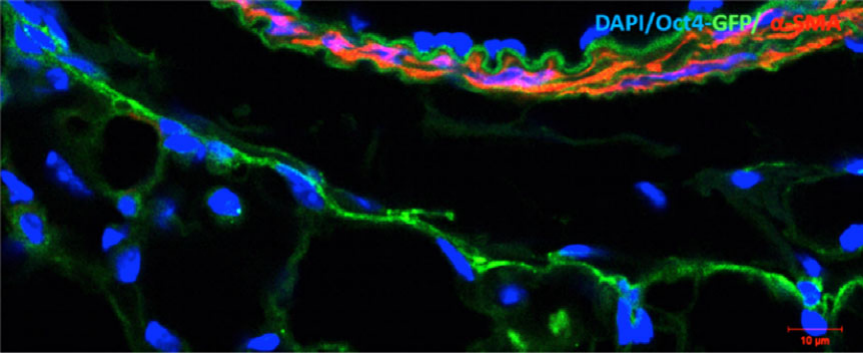
Representative confocal images of Oct4-positive cells based on GFP fluorescence in Oct4-GFP transgenic mouse lungs. This image shows that Oct4 is confined to the adventitial cells of a blood vessel. The cells also present long extensions which tend to establish intercellular contacts. Shown in red is α-SMA labelling of the vascular smooth muscle cells. Bar = 10 μm.

Immunoconfocal analysis of lung tissues in Oct4-GFP transgenic mice showed that the cells expressing Oct4 display TC features. Oct4-GFP^pos^ cells showed long extensions and exhibited a strong tendency to create cell-to-cell contacts between them (Fig. 3).

### Oct4-positive cells display TC features in culture

**Fig. 4:**
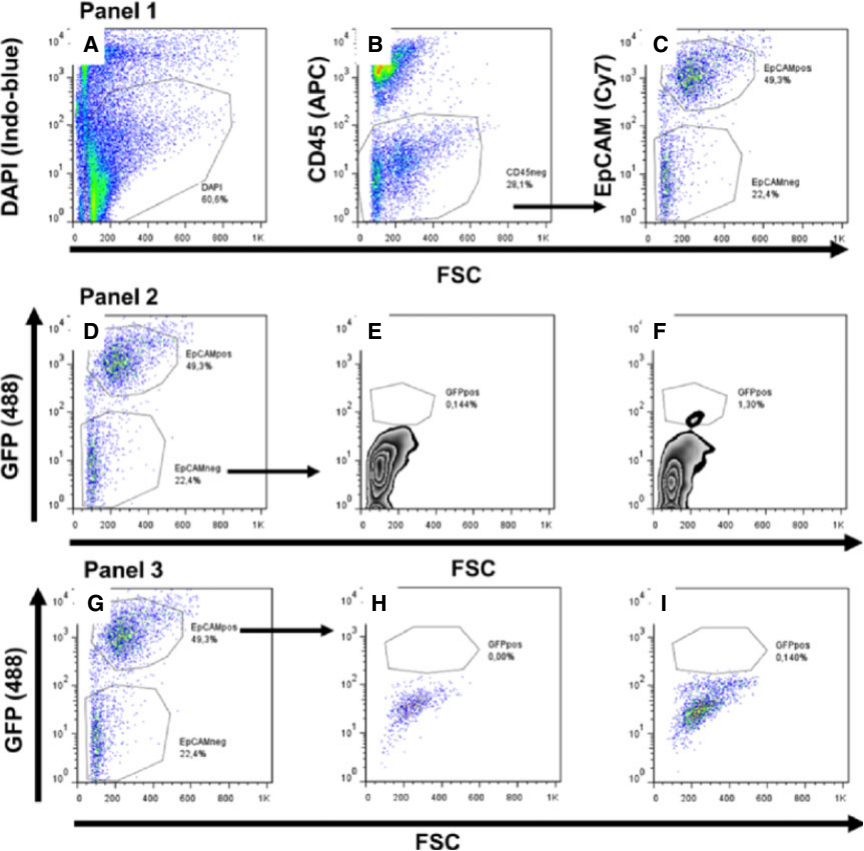
Flow cytometry analysis of lung cells isolated from adult Oct4/GFP transgenic mice. Panel 1: (**A**) Exclusion of dead cells. Viable cells are DAPI negative and dead cells are DAPI positive. (**B**) Exclusion of CD45^pos^ hematopoietic cells and gating for CD45^neg^ cells. (**C**) Chosen settings for CD45^neg^ cells in EpCAM^pos^ and EpCAM^neg^ cell population. Panel 2: (**D**) Identical settings as shown in **C** where EpCAM^neg^ cells, when compared to the negative control (**E**) contains a small population of EpCAM^neg^CD45^neg^EpCAM^neg^GFP^pos^ cells representing only 1.3% from the total EpCAM^neg^ cells (**F**). Panel 3: (**G**) Identical settings as shown in **C**, where EpCAM^pos^ cell population, when compared to negative controls (**H**) contains only a few GFP-positive (EpCAM^neg^CD45^neg^EpCAM^neg^GFP^pos^) cells (**I**). Unstained cell suspensions from wild-type were mice used as negative controls (**E** and **H**).

Oct4-positive cells were sorted based on GFP fluorescence, and the cell population EpCAM^neg^/CD45^neg^/Oct4-GFP^pos^ was identified (Fig. 4). Dead cells were excluded with DAPI (Fig. 4A). Hematopoietic CD45^pos^ cells were excluded from the analysis as well (Fig. 4B). EpCAM^pos^ epithelial cells and EpCAM^neg^ cells were separately analysed (Fig. 4C). A small population (1.3%) of Oct4-GFP^pos^ cells was identified in the EpCAM^neg^ cell fraction (Fig. 4D through F, panel 2). Conversely, Oct4-GFP^pos^ cells were almost absent in the EpCAM^pos^ cell fraction (Fig. 4G through I, panel 3).

**Fig. 5:**
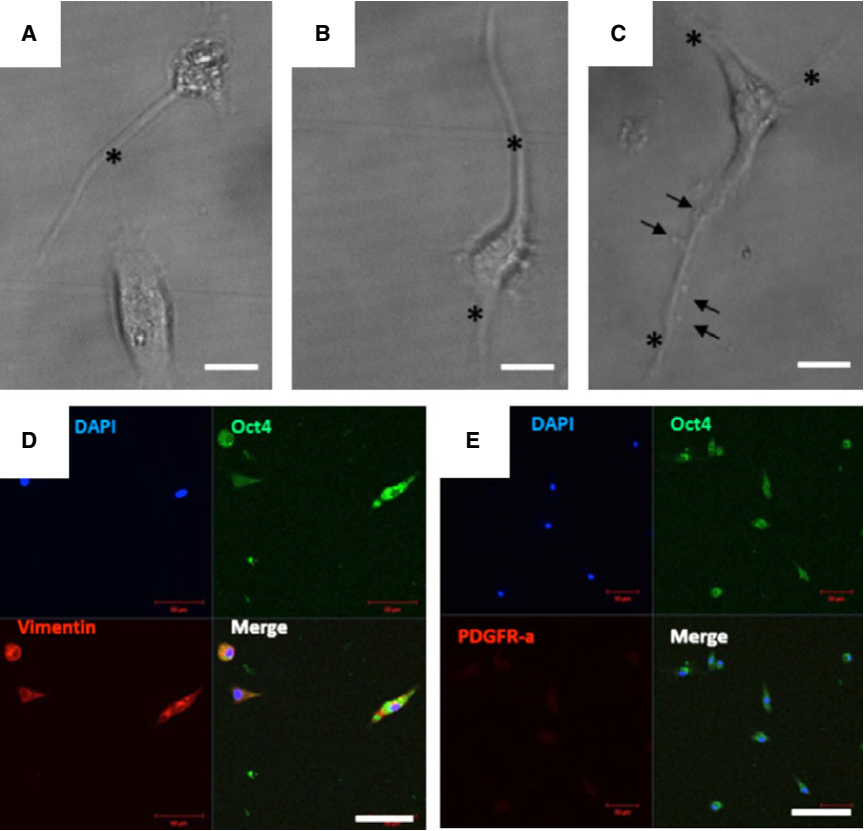
Isolated Oct4-GFP^pos^ cells and maintained in culture for 5 days. Cells were sorted based on GFP fluorescence and kept in culture at 37°C in MEM medium. (**A**–**C**) Bright field images show cells with a fusiform telocyte-like shape with thin prolongations (asterisks). Examples are provided for cells having one (**A**), two (**B**) or three (**C**) cell prolongations (telopodes) with a particular shape resembling a string of beads (arrows in **C**). (**D**) Co-expression of Oct4 and vimentin in telocytes. (**E**) Co-staining of telocytes with Oct4 and PDGFR-α. Note that telocytes do not express PDGFR-α; bar = 50 μm

After cell sorting, the Oct4/GFP^+^ cells were plated and kept in culture under normoxic conditions. After 5 days in culture, the cells showed a fusiform shape resembling that of TCs with one (Fig. 5A), two (Fig. 5B) or three (Fig. 5C) extensions. In addition, cells with moniliform extensions were conspicuously observed (Fig. 5C). The immunofluorescence staining of Oct4-positive cells showed that these cells were positive for vimentin (Fig. 5D) and negative for PDGFR-α (Fig. 5E).

### Oct4-positive cells express several TC markers mainly in the perivascular areas

**Fig. 6:**
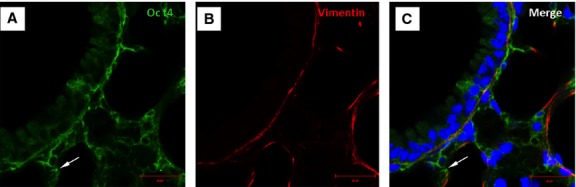
Immunofluorescent confocal images of Oct4 and vimentin in bronchus. (**A**) Oct4-positive cells are localized in a bronchus between the bronchiolar epithelium and smooth muscle cells layer. (**B**) Immunolabeling for vimentin. (**C**) The merged picture shows co-localization of Oct4 and vimentin. Note that Oct4-positive cells extend to the alveolar septum (arrow); bar = 10 μm.

**Fig. 7:**
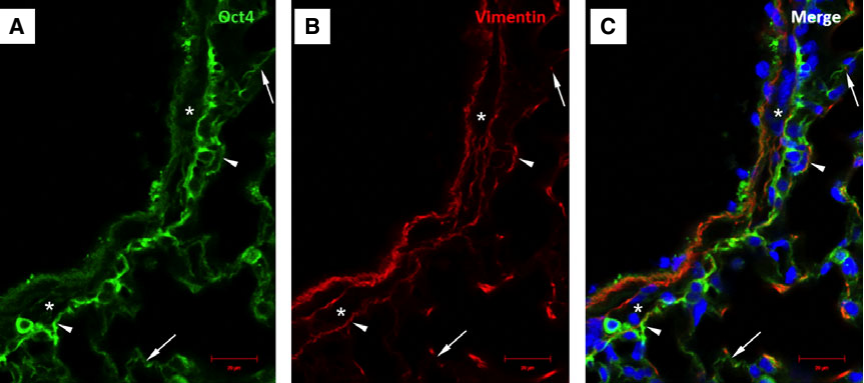
Immunofluorescence of Oct4 and vimentin in the vascular wall and perivascular regions. (**A**) Oct4-positive cells circumscribe the smooth muscle cell layer (asterisk). (**B**) Immunolabeling for vimentin. (**C**) Arrowheads in the merged picture indicate cells which are positive for both, Oct4 and vimentin. Note that Oct4-positive cells extend to the alveolar septum (arrows); bar = 10 μm.

**Fig. 8:**
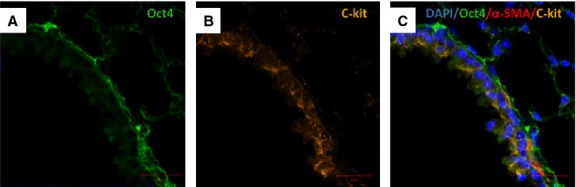
Immunofluorescent confocal images of Oct4 and C-kit labelling pattern in bronchus. (**A**) Oct4-positive cells are present in the peribronchial area. (**B**) Some epithelial cells are positive for c-kit. (**C**) The merged picture demonstrates little co-localization of Oct4 with C-kit. α-SMA labelling (red) of smooth muscles lining the bronchiolar epithelium; bar = 10 μm.

**Fig. 9:**
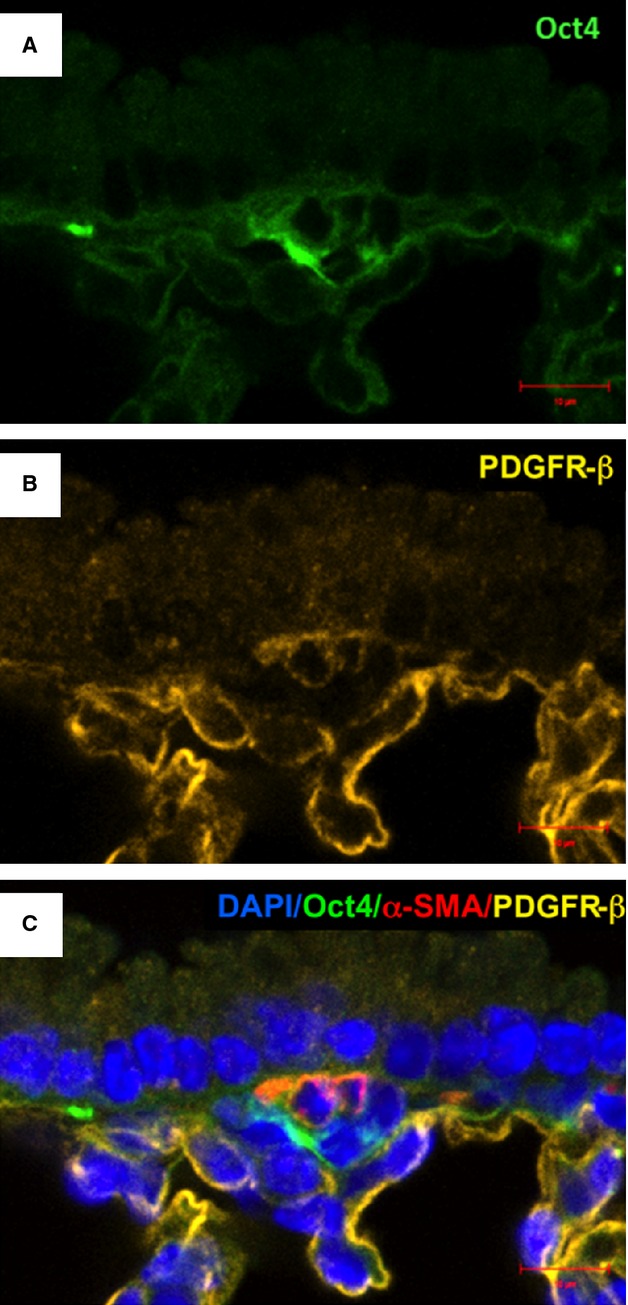
Immunofluorescent confocal images of Oct4 and PDGFR-β in bronchus. (**A**) Oct4-positive cells are located beneath the bronchial epithelium. (**B**) PDGFR-β labels more bronchial cells than Oct4. (**C**) The merged picture demonstrates that the two markers weakly co-localize. α-SMA stains the smooth muscles lining the bronchiolar epithelium (**C**); bar = 10 μm.

**Fig. 10:**
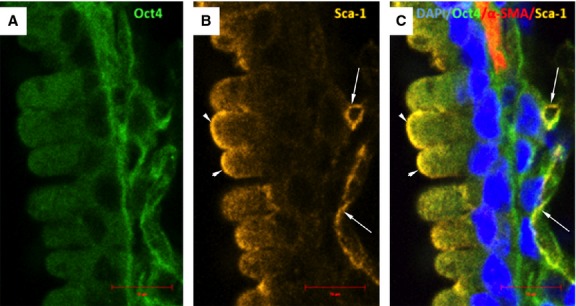
Confocal images of Oct4 and Sca-1 immunolabeling in bronchus. (**A**) Oct4-positive cells are located in close vicinity with a bronchus. (**B**) Sca-1 is confined to some bronchilar epithelial cells (arrowheads) or peribronchial cells (arrows) where co-localizes with Oct4. (**C**) Arrows in the merged picture indicate the cells showing co-localization of Oct4 and Sca-1. Nuclei are stained blue with DAPI, and smooth muscle cells are labelled in red with α-SMA; bar = 10 μm.

**Fig. 11:**
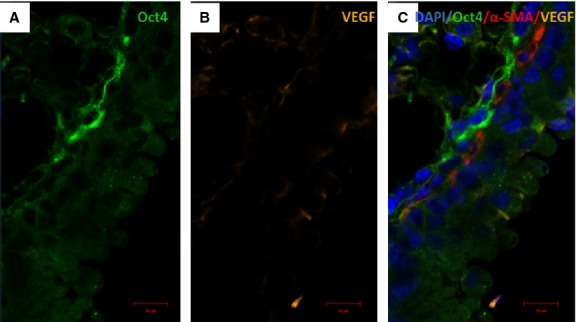
Co-staining of Oct4 and VEGF in bronchus. (**A**) Oct4-positive cells are located in the peribronchial areas. (**B**) VEGF is modestly expressed in bronchial cells. (**C**) The merged picture demonstrates that the two markers co-localize very weak. (**C**) α-SMA labelling is shown in red; bar = 10 μm.

**Fig. 12:**
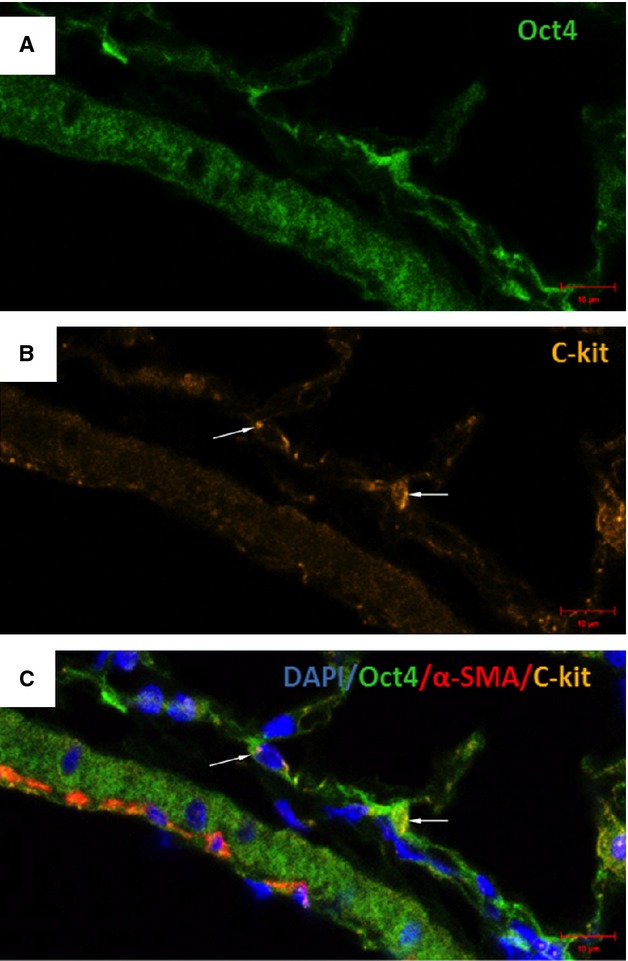
Co-staining of Oct4 and C-kit in blood vessels. (**A**) Oct4-positive cells are present in the adventitia of blood vessels. (**B**) C-kit also labels some perivascular cells (arrows). (**C**) The merged picture displays cells with co-localization of Oct4 and C-kit (arrows); bar = 10 μm.

**Fig. 13:**
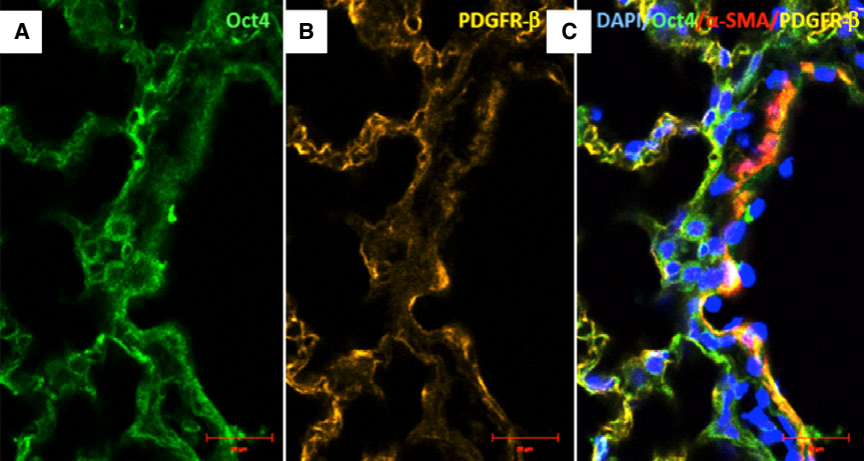
Immunofluorescence of Oct4 and PDGFR-β in blood vessels. (**A**) Numerous Oct4-positive cells are seen in the adventitia. (**B**) PDGFR-β is also expressed in perivascular cells. (**C**) The merged picture demonstrates that Oct4 co-localizes with PDGFR-β; bar = 10 μm.

**Fig. 14:**
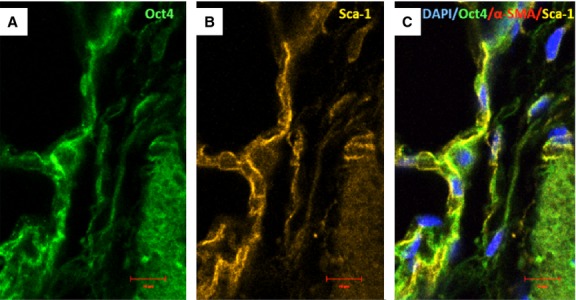
Immunofluorescence of Oct-4 and Sca-1 in blood vessels. (**A**) Oct4-positive cells are present in the perivascular areas and in the vascular wall. (**B**) Sca-1 is also expressed in such areas. (**C**) The merged picture demonstrates co-localization of Oct4 and Sca-1. α-SMA (red) labels vascular smooth muscle cells (**C**); bar = 10 μm.

**Fig. 15:**
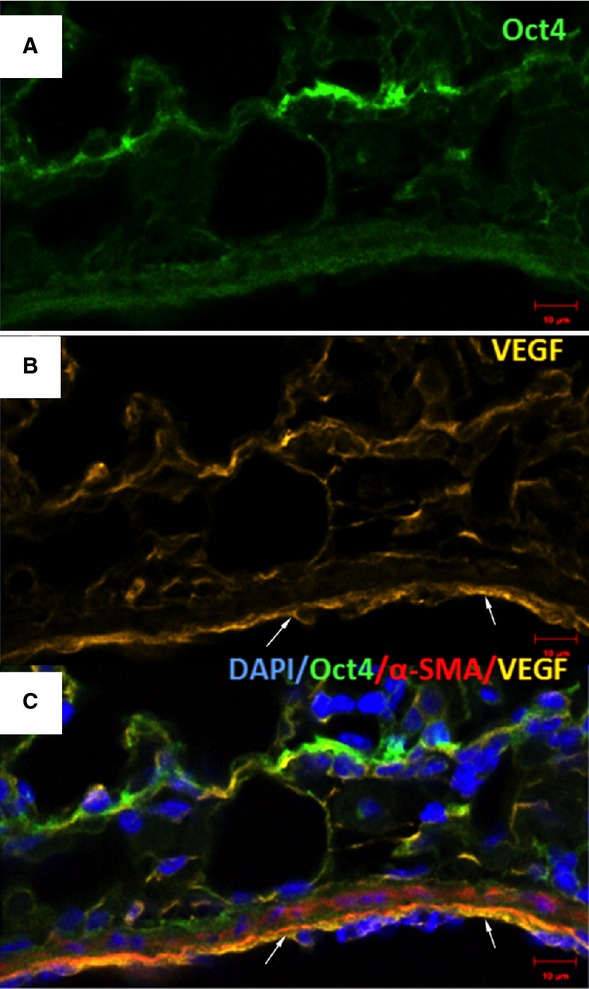
Immunofluorescence of Oct4 and VEGF in blood vessels. (**A**) Oct4-positive cells are located in the perivascular areas and co-localize with VEGF (B). Notice abundant VEGF expression in endothelial cells (arrows). (**C**) The merged picture demonstrates co-localization of Oct4 and VEGF. α-SMA (red) labels smooth muscle cells in the media; bar = 10 μm.

Immunofluorescent confocal analyses carried out on paraffin-embedded tissues showed that Oct4-positive cells expressed several described markers of TCs including vimentin, C-kit, PDGFR-β, Sca-1 and VEGF. Our data showed that Oct4 co-localized with vimentin in both, peribronchial (Fig. 6) and perivascular regions (Fig. 7). In the peribronchial/peribronchiolar areas we found very little co-localization of Oct4 with C-kit (Fig. 8), PDGFR-β (Fig. 9), Sca-1 (Fig. 10) and VEGF (Fig. 11). In contrast, the adventitia of blood vessels showed a marked co-localization of Oct4 with C-kit, PDGFR-β, Sca-1 and VEGF (Figs 12–15).

### Gene expression of TC markers in adult mouse lung tissues

**Fig. 16:**
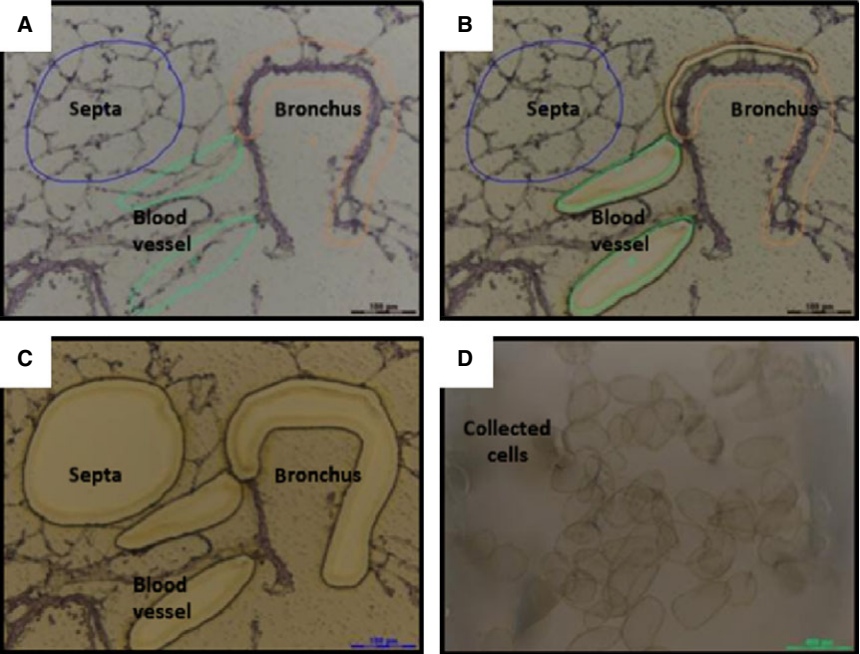
Sequential steps of laser capture microdissection of mouse lung cryosections. (**A**) Before capture, the regions of interest (ROI) are selected. (**B**) During the capture, laser sectioning of ROI. (**C**) After capture, the ROI are collected. (**D**) Image showing dissected ROI.

**Fig. 17:**
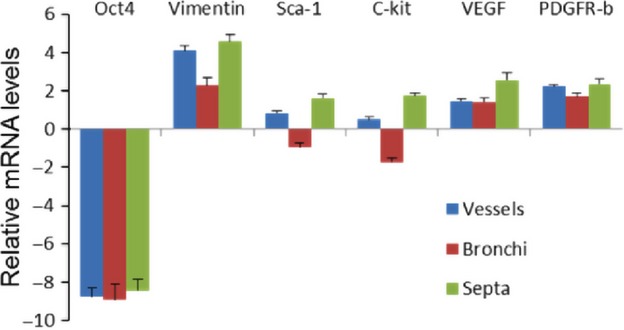
mRNA expression levels of telocyte markers after cell picking by laser capture microdissection. The expression levels of Oct4, vimentin, Sca-1, C-kit, VEGF and PDGR-β were determined by qRT-PCR. Cells from different locations including peribronchial and perivascular regions and alveolar septa were selected by laser capture microdissection and the RNA was isolated. Note that Oct4 expression is relatively low and is almost similar in all ROI, whereas vimentin, C-kit and Sca-1 mRNA in vessels and alveolar septa are higher than in bronchi. VEGF is higher in septa, whereas the levels of PDGFR-β were similar in all compartments. Data are expressed in ΔCT values normalized with GAPDH housekeeping gene control.

Single cell picking was performed with laser capture microdissection technique at the TC locations. After the dissection (Fig. 16A–C), the cells were collected (Fig. 16D) and the RNA was then isolated. The gene expression levels of the TC markers were determined by real-time quantitative PCR. The data showed that the Oct4 mRNA levels, albeit very low, were similar in vessels, bronchi, and septa. The vimentin, C-kit and Sca-1 mRNA levels were higher in vessels and alveolar septa than in bronchi. The VEGF mRNA expression was the highest in septa, whereas the mRNA levels of PDGFR-β were similar in all three compartments (Fig. 17).

### Pluripotency-related genes in Oct4-positive cells and ESCs

**Fig. 18:**
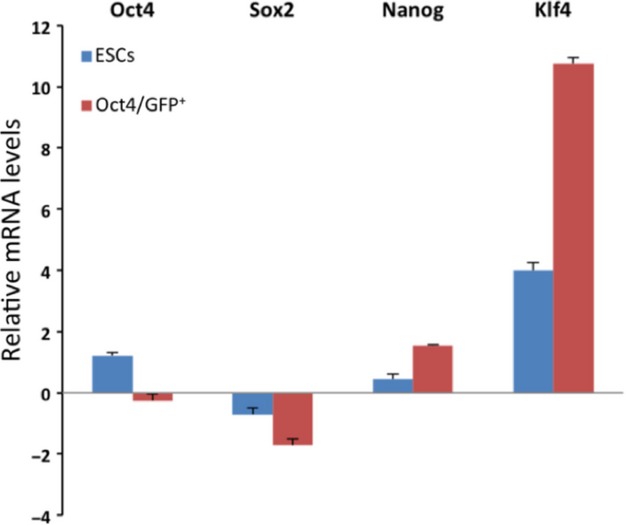
Oct4, Sox2, Nanog and Klf4 mRNA expression levels in Oct4-positive cells and mouse embryonic stem cells. Data are expressed in ΔCT values normalized with GAPDH housekeeping gene control.

Mounting evidence indicates that maintenance, self-renewal and pluripotency of embryonic or adult stem cells is regulated by several transcription factors such as Nanog, Sox2 and Klf4 [28–32]. To investigate whether Oct4-positive cells possess a regulatory network similar to that of ESCs we have conducted qRT-PCR analysis of Oct4, Sox2, Nanog and Klf4 transcripts in Oct4-positive cells and mouse ESCs. The results presented in Figure 18 show that Oct4 and Sox2 transcripts in Oct4-positive cells is lower than in ESCs. However, the mRNA levels of Nanog and Klf4 was found to be higher in Oct4-positive cells than in ESCs. These data indicate that Oct4-positive cells express a subset of pluripotency-related genes that are also expressed in pluripotent ESCs [33,34].

### Ultrastructure of lung TCs

**Fig. 19:**
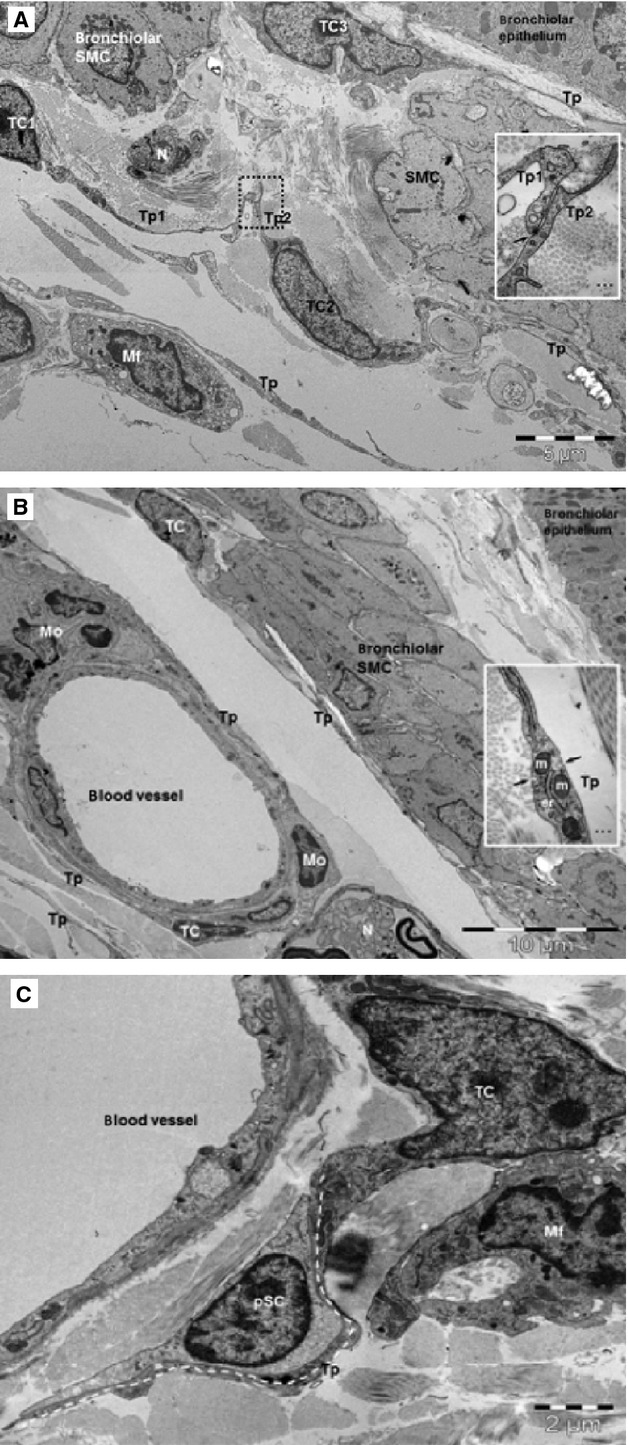
TEM images show telocytes (TCs) with telopodes (Tp) in mouse lung. (**A**) TCs form a peribronchiolar network beneath the epithelium and muscular layer (SMC: smooth muscle cells). Inset: Puncta adhaerentia junction (arrow) connects the telopodes (Tp1, Tp2) of different TCs. Mf: macrophage; N: nerve endings. (**B**) TCs and Tp surround blood vessel and line the bronchiolar SMC. N: nerve; Mo: mononuclear cells. Inset: higher magnification of a telopode (Tp) shows caveolae (arrows), mitochondria (m) and endoplasmic reticulum. (**C**) A TC in the perivascular space has close contact (stromal synapse) with a putative stem cell (pSC) in a terminal mouse bronchiole. pSC are undifferentiated cell which have scanty cytoplasm, very few ER cisternae and numerous ribosomes. TC has a telopode (Tp) around a pSC (dotted line).

Transmission electron microscopy showed a network of TCs located beneath bronchiolar epithelium (Fig. 19A), surrounding bronchiolar muscle cells (Fig. 19A and B), and blood vessels (Fig. 19B). Small adherens junctions were found to connect the TCs in this network (Fig. 19A inset). Telocytes showed characteristic ultrastructural features: a thin layer of cytoplasm around nucleus and long telopodes, and cellular prolongations with a narrow emergence from the cellular body (Fig. 19A and B). Telopodes showed very long slender segments (50–150 nm) and dilation (about 500 nm) containing mitochondria and endoplasmic reticulum (Fig. 19B, inset). Telocytes were found to have close contacts with undifferentiated putative stem cells in the perivascular space (Fig. 19C).

## Discussion

The purpose of this study was to characterize and trace Oct4-positive cells in adult mouse lung. The expression of Oct4 in adult tissues is indeed a controversial topic in the field of adult stem cells research [27,29,30,35]. The presence of Oct4 in adult tissue has been indeed related to either PCR product artefacts, Oct4 pseudogenes or to other members of Oct family [13,35,36]. In the present study, we have demonstrated that Oct4 was expressed in adult mouse lung tissues. Oct4 expression was first detected by western blot and immunohistochemical examination of lung tissue sections and revealed that Oct4-positive cells were located in the peribronchial/peribronchiolar and perivascular spaces. Moreover, we have used Oct4-GFP transgenic mice which revealed a similar localization of the Oct4-GFP signal with that of DAB. The localization of Oct4-positive TCs detected in wild-type or Oct4-GFP transgenic mice were confirmed by electron microscopy. In fact, several recent studies also revealed that TCs are present in the murine pulmonary tissues lining blood vessels and bronchi [20].

In this study, we were able to isolate Oct4-positive cells and maintain them in culture. It is worthy to note that Oct4-positive cells *in vitro* displayed similar structural features with already described characteristics of TCs, isolated from various organs [16,21,37]. In particular, we found similar numbers and shapes of telopodes growing and extending from Oct4-positive cells. These results are in accordance with the observations in isolated and cultured Sca-1 TCs [21,38]. Moreover, we observed that Oct4-positive cells exhibit moniliform prolongations originating from the cell body. These features are also typical for TCs [38].

We have also identified Oct4-positive cells in the peripheral mononuclear blood cell population but we did not focused on this cell type. They were discriminated during the isolation with an anti-CD45 antibody and therefore, only the EpCAM^neg^/CD45^neg^/Oct4-GFP^pos^ cell fraction was characterized. Moreover, immunostaining of isolated and cultured Oct4-GFP^pos^ cells showed that these cells were positive for vimentin and negative for PDGFR-α, a marker that is more representative of fibroblast cell lineages. Thus, these data provide another evidence that Oct4-positive cells are TCs having mesenchymal origin by expressing vimentin but they are not fibroblasts because of the lack of PDGFR-α expression. Our results are in agreement with studies comparing the gene expression profile of TCs to that of fibroblasts in murine lung and clearly demonstrate that TCs are not fibroblasts [39]. In addition, numerous studies have demonstrated that TCs have different ultrastructure and phenotype than fibroblasts (for review see: [17,21]). Moreover, the present study based on negative selection with EpCAM, provide additional evidence that EpCAM^neg^/CD45^neg^/Oct4-GFP^pos^ cells are not epithelial cells but represent a subtype of Oct4-positive TCs.

By immunofluorescence, we confirmed that Oct4-positive cells share several markers with TCs including C-kit, PDGFR-β, Sca-1 and VEGF. It is important to mention that such a co-localization was mainly observed in the perivascular areas, while in the peribronchial/peribronchiolar spaces showed little co-localization. These observations suggest that TCs in the lung represent, at least immunophenotypically, a heterogenous cell type. The expression of the different above mentioned markers were confirmed on the mRNA level by qRT-PCR of cells captured with laser microdissection from different locations including blood vessels, bronchi and alveolar septa. It is obvious that the mRNA levels of the above mentioned TC markers do not fully concur with the immunohistochemical analyses. This especially applies to the peribronchial TCs. However, it is possible that such a difference in the expression profile might be because of the contamination with other Oct4-negative cells which might have a different expression profile.

In summary, we were able to show that Oct4 is expressed in adult mouse lung. In addition, we have demonstrated that Oct4-positive cells closely resemble the morphology of TCs seen by electron microscopy. It is concluded for the first time that TCs in adult lung mouse tissue comprise Oct4-positive cells which express pluripotency-related genes and represent therefore a population of adult stem cells which might contribute to lung regeneration.
